# Stabilizing Halide Distribution in Mixed Halide Perovskites through Diammonium-Based Passivation

**DOI:** 10.1021/acsomega.6c01620

**Published:** 2026-03-16

**Authors:** Amalraj Peter Amalathas, Saisankar Sunthareswaran, Neda Neykova, Lukáš Horák, Jakub Holovský

**Affiliations:** † Department of Physics, Faculty of Science, 70709 University of Jaffna, Jaffna 40000, Sri Lanka; ‡ SOL-MAT Lab (Solar Cell Material laboratory), Faculty of Electrical Engineering, 86889 Czech Technical University in Prague, Technická 2, Prague 166 27, Czech Republic; § Institute of Physics, Czech Academy of Sciences, v. v. i., Cukrovarnická 10, Prague 162 00, Czech Republic; ∥ Department of Condensed Matter Physics, Faculty of Mathematics and Physics, Charles University, Ke Karlovu 5, Prague 2 12116, Czech Republic

## Abstract

Mixed halide perovskite solar cells (PSCs) are promising for high-efficiency tandem photovoltaic architectures, but their performance is hindered by light-induced halide segregation and defect-mediated nonradiative recombination. Here, we introduce 1,3-diaminopropane dihydroiodide (PDADI) as a multifunctional surface passivation material to simultaneously suppress halide migration and reduce defect densities in FA_0.83_Cs_0.17_Pb­(I_0.6_Br_0.4_)_3_ wide bandgap (∼1.78 eV) perovskite films. Our investigations reveal that PDADI-treated films exhibit a substantial reduction in light-induced phase segregation, with the photoluminescence (PL) peak shift suppressed under continuous illumination, an effect further confirmed by UV–vis absorption spectroscopy. Fourier-transform infrared analysis shows that PDADI’s terminal −NH_3_^+^ groups engage in strong bidentate coordination with undercoordinated Pb^2+^ ions, while its iodide counterions help compensate halide vacancies. This dual interaction stabilizes the perovskite lattice, reducing the trap density as evidenced by space-charge-limited current measurements. Consequently, trap-assisted nonradiative recombination is reduced, resulting in enhanced PL intensity and longer carrier lifetimes. PSCs incorporating PDADI passivation achieve a notable improvement in power conversion efficiency, increasing from 14.11% to a champion value of 16.54%, driven by an increase in open-circuit voltage from 1.135 to 1.243 V, and a fill factor improvement from 68.8% to 72.7%. Additionally, *J*–*V* hysteresis is significantly reduced from 9.6% to 3.6%, indicating improved charge extraction and suppressed ion migration. These results highlight the potential of PDADI as an effective molecular passivator for enhancing the performance and stability of wide bandgap mixed halide PSCs, advancing their applicability in tandem solar cell technologies.

## Graphical abstract

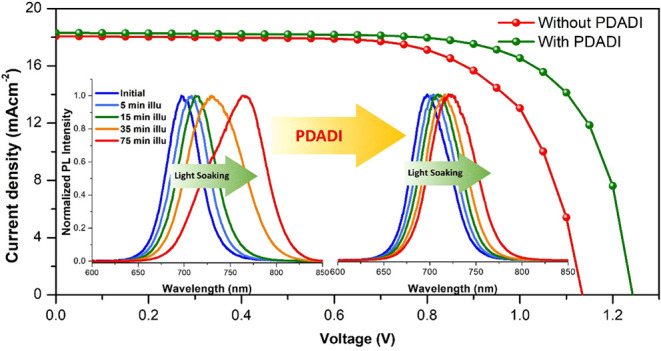

## 1 Introduction

Metal halide perovskite solar cells (PSCs) have achieved rapid efficiency improvements, with certified power conversion efficiencies (PCEs) reaching up to 27% for single-junction devices and up to 34.9% in perovskite-silicon tandem configurations.^1^ The strategic importance of wide bandgap perovskites is paramount for achieving these high efficiencies in tandem configurations. Optimal performance in perovskite-silicon tandem cells, for instance, critically relies on integrating a wide bandgap (∼1.7–1.9 eV) perovskite as the front cell, which is then paired with a low bandgap absorber.^2^ It is important to note that while record efficiencies for narrow-bandgap materials exceed 27%, the performance of wide-bandgap (WBG) absorbers is inherently lower due halide distribution stability under illumination (bandgap ∼ 1.72 eV) utilized in this work, recent benchmarks report a record efficiency of approximately 17.1%.^3^ The efficiency gap between these WBG materials and their narrow-bandgap counterparts is primarily driven by loss channels such as nonradiative recombination at grain boundaries and halide demixing under operational stress. Among various compositions, formamidinium-cesium lead iodide-bromide compositions (FA_1–*x*_Cs_*x*_Pb­(I_1–*y*_Br_*y*_)_3_) are particularly promising due to their tunable bandgaps and improved thermal stability, achieved by eliminating volatile methylammonium (MA^+^) cations.^4^ Despite their immense promise, these mixed halide perovskites tend to separate into bromide-rich and iodide-rich domains under illumination, a phenomenon widely recognized as light-induced halide segregation.^5,6^ This intrinsic instability leads to adverse effects such as bandgap fluctuations, photoluminescence red shifts, and voltage losses.^7–10^ Ultimately, such halide distribution instability undermines the efficiency of wide bandgap PSCs and significantly limits their potential in advanced tandem architectures. Therefore, maintaining a homogeneous mixed halide perovskite composition under operating conditions and effectively suppressing halide segregation are essential for achieving both high initial efficiency and reliable device performance.

One root cause of halide segregation is the presence of defects and local halide distribution inhomogeneities, which act as pathways or nucleation sites for ion migration.^7,11–13^ Specifically, undercoordinated lead atoms and halide vacancies, particularly located on the perovskite surface or at grain boundaries, can initiate and accelerate phase separation. These defects also contribute to increased nonradiative recombination, further deteriorating device performance.^14^ Recent studies have highlighted that light-induced ion migration and subsequent halide-distribution changes are the primary degradation pathways for high-bromide WBG cells.^15^ In response to the challenges posed by defects and instabilities, surface passivation strategies have emerged as a highly effective approach to simultaneously reduce defect-assisted recombination and improve the halide-distribution stability of mixed halide perovskites.^16–18^ Perovskite films produced via solution-processing methods are typically polycrystalline, characterized by numerous grain boundaries and interfaces that are rich in deep energy level defects.^19^ These defects, which include atomic vacancies, interstitials, and dislocations at grain boundaries, introduce detrimental energy levels within the perovskite’s electronic structure.^20^ Their presence negatively impacts PSC performance by diminishing steady-state charge density, reducing quasi-Fermi level splitting, and consequently lowering both the open-circuit voltage (Voc) and fill factor (FF).^21–23^

The incorporation of organic cations serves as a prominent strategy for surface passivation.^24–28^ These cations can effectively cap the perovskite surface, healing undercoordinated lead sites and providing anions to fill halide vacancies, thereby mitigating both electronic traps and ion migration pathways. Diammonium cations have recently gained attention as an effective passivation for wide bandgap mixed halide perovskites.^29–31^ Unlike monofunctional ammoniums, diammonium molecules contain two −NH_3_^+^ groups that can interact with the perovskite surface at two points, potentially forming a Dion–Jacobson 2D perovskite layer or bidentate bonds across undercoordinated sites.

While various diammonium salts have been explored for surface passivation, PDADI (1,3-diaminopropane dihydroiodide) offers distinct structural advantages. Unlike rigid aromatic diammonium systems such as 1,4-phenylenediammonium (PDA),^32^ which can impose steric constraints and potentially lead to the formation of resistive low-dimensional phases, PDADI features a flexible aliphatic propane chain.^33^ This flexibility allows for a more conformal bidentate coordination to the perovskite surface, effectively anchoring undercoordinated Pb^2+^ and halide ions. This structural adaptability ensures that PDADI matches or exceeds the passivation effects of rigid systems by providing a more uniform protective shell that inhibits ion migration without hindering charge transport.

Here, we introduce PDADI as a multifunctional passivation material for wide-bandgap FA_0.83_Cs_0.17_Pb­(I_0.6_Br_0.4_)_3_ perovskite films. This study proposes that PDADI provides a synergistic passivation effect by anchoring to undercoordinated lead sites via its bidentate ammonium groups while simultaneously compensating halide vacancies through its iodide anions. This dual interaction is expected to reduce nonradiative recombination and inhibit halide ion migration. We demonstrate that applying a PDADI treatment leads to a significant improvement in optoelectronic quality and device performance. The PDADI-treated perovskite maintains a stable single-phase halide-distribution under illumination, indicating effective suppression of phase segregation. Furthermore, PDADI modification results in higher photovoltaic efficiency, with notable improvements in Voc and halide-distribution stability compared to untreated control films. These findings highlight the effectiveness of PDADI as a surface passivation strategy for bromide-rich mixed-halide perovskites, supporting the development of wide-bandgap absorbers with improved halide-distribution stability for high-efficiency tandem solar cell applications.

## 2 Results and Discussion

To systematically examine the optical changes induced by passivation and to elucidate the role of light-induced phase segregation on halide distribution, PL spectroscopy was employed. This technique, highly sensitive to variations in a material’s electronic band structure and defect landscape, serves as an ideal diagnostic tool for evaluating the stability of mixed-halide perovskites. Both PDADI passivated and unpassivated samples were subjected to continuous 1 Sun illumination using a simulated solar light source, and PL spectra were recorded at defined time intervals. Figure 1a shows the normalized PL spectra of the control perovskite film (without PDADI passivation) under different illumination durations. The fresh film initially exhibits a prominent emission peak centered around 697 nm, as expected for the unsegregated FA_0.83_Cs_0.17_Pb­(I_0.6_Br_0.4_)_3_ perovskite composition. Upon illumination, the peak undergoes a rapid and substantial redshift, progressing from 697 to 765 nm over 75 min. This pronounced shift is a characteristic indicator of light-induced halide phase segregation in mixed halide perovskites. During illumination, mobile halide ions (I^–^ and Br^–^) migrate and segregate into thermodynamically favored iodide-rich and bromide-rich domains. The resulting iodide-rich regions having a lower bandgap dominate the PL signal and act as nonradiative recombination centers, reducing charge carrier collection and contributing to lower open-circuit voltages in devices.^7^ Furthermore, the PL spectrum recorded after 40 h of dark storage shows only partial recovery. This suggests that the light-induced halide segregation in the control films involves high kinetic barriers for ion back-migration, leading to a persistent nonuniformity in the halide distribution, rather than a fully reversible process.

**Figure 1:**
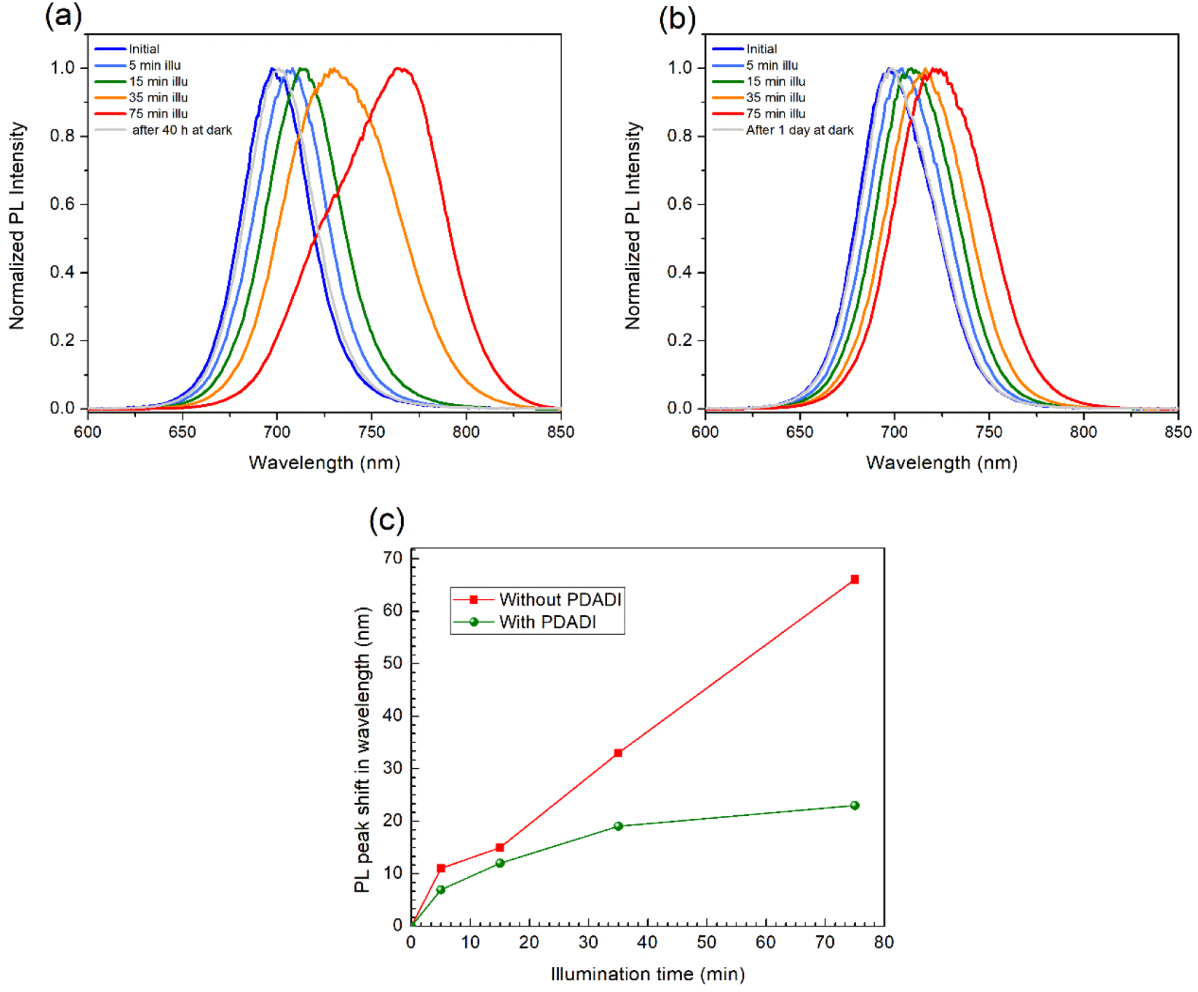
Normalized photoluminescence (PL) spectra of (a) control FA_0.83_Cs_0.17_Pb­(I_0.6_Br_0.4_)_3_ perovskite film and (b) PDADI-passivated FA_0.83_Cs_0.17_Pb­(I_0.6_Br_0.4_)_3_ perovskite film, measured under continuous 1 Sun illumination (0, 5, 15, 35, and 75 min) and after dark recovery. (c) Comparison of the PL peak shift as a function of illumination time for perovskite film without and with PDADI passivation.

In contrast, Figure 1b shows the normalized PL spectra of the perovskite film treated with PDADI. A remarkable stabilization of the PL peak position is observed. Although a slight redshift occurs initially, its magnitude and progression under illumination are significantly reduced. After 75 min of continuous illumination, the PL peak shifts only to 720 nm, indicating substantial resistance to halide demixing. Furthermore, dark recovery is notably more effective in PDADI treated films than in the control films, implying that any light-induced segregation is either less severe or more kinetically reversible. These results highlight the effectiveness of PDADI in preserving halide homogeneity under illumination stress. To quantitatively highlight this difference, Figure 1c compares the PL peak shift relative to the initial emission wavelength for both control and PDADI passivated films as a function of illumination time. The control sample exhibits a rapid and nearly linear redshift, exceeding 65 nm after 75 min of illumination. This continuous and pronounced shift reflects extensive light-induced halide migration and progressive phase segregation, confirming the dynamic instability of the mixed halide perovskite under photoexcitation. In contrast, the PDADI-treated film demonstrates a significantly reduced initial redshift; more importantly, the rate of the shift decreases substantially after approximately 15–35 min of illumination. Beyond this point, the redshift stabilizes at approximately 23 nm, revealing a distinct plateau behavior. This plateau is a clear indication that PDADI passivation not only delays the onset of halide segregation but also effectively constrains its progression, limiting the extent of ion migration even under continuous stress. Such stabilization suggests that the PDADI treatment successfully establishes improved long-term kinetic stability by mitigating the driving forces and pathways for halide ion migration. Furthermore, the notably more effective dark recovery in PDADI-treated films implies that any light-induced changes are either less severe or more kinetically reversible than in the control films.

To further validate the PL observations and gain deeper insights into the macroscopic halide distribution evolution under illumination, UV–visible absorption spectroscopy was employed. Absorbance spectra of both unpassivated and PDADI passivated perovskite samples were recorded at various time intervals under continuous illumination, as shown in Figure 2a and b, respectively. To highlight subtle spectral changes, differential absorbance spectra (Δ*A*) were calculated by subtracting the initial spectrum from those obtained at later time points, and are presented in Figure 2c and d. Figure 2a displays the evolution of the absorption spectrum for the control film during illumination. Initially, the film exhibits a sharp absorption edge around 680–690 nm, characteristic of the mixed halide composition. However, with prolonged light exposure, significant spectral changes indicative of halide segregation emerges. A noticeable reduction in absorbance is observed in the 630–680 nm region, corresponding to the depletion of the original mixed halide phase. Simultaneously, a new absorption feature appears and intensifies beyond 700 nm, signaling the formation of iodide-rich domains with narrower bandgaps. These observations align with the redshift behavior seen in the PL spectra and signify a progressive phase segregation process. This trend is more clearly illustrated in the Δ*A* spectra for the control film in Figure 2c. A distinctive bipolar pattern emerges, characterized by a negative Δ*A* peak in the shorter wavelength region, representing loss of the mixed halide phase, and a positive Δ*A* peak in the longer wavelength region, associated with the emergence of iodide-rich segregated domains. The magnitude of both peaks increases steadily with illumination time, clearly confirming the continuous and cumulative nature of halide migration in the absence of passivation.

**Figure 2:**
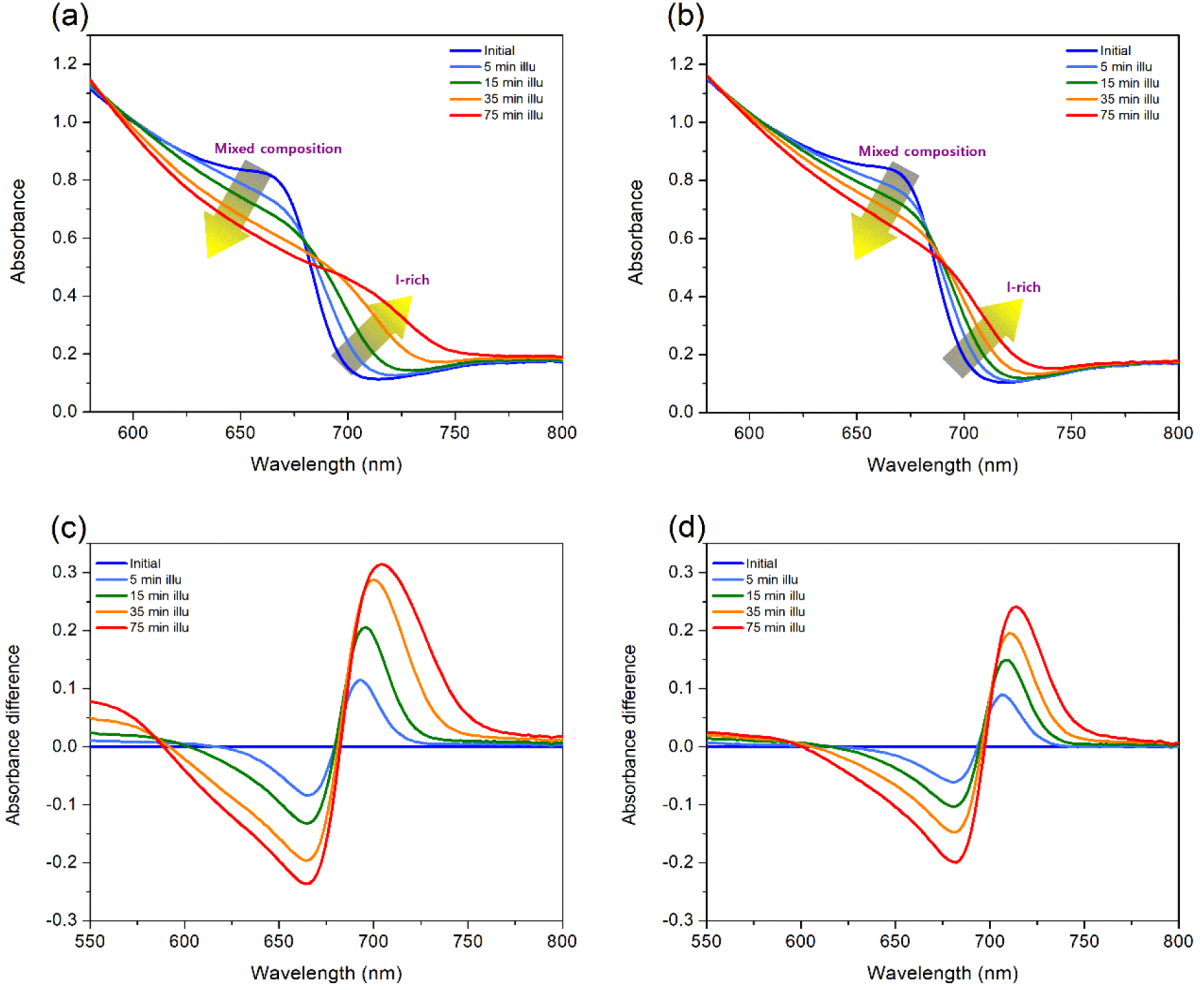
UV–vis absorbance spectra of (a) unpassivated (control) and (b) PDADI passivated FA_0.83_Cs_0.17_Pb­(I_0.6_Br_0.4_)_3_ perovskite films under continuous 1 Sun illumination for various durations (initial, 5, 15, 35, and 75 min). Corresponding differential absorbance (Δ*A*) spectra, obtained by subtracting the initial spectrum from each subsequent spectrum, are shown for (c) the control film and (d) the PDADI passivated film.

In contrast, the PDADI passivated film, shown in Figure 2b, demonstrates much greater spectral stability under the same illumination conditions. The development of iodide-rich features is significantly suppressed. Only minor variations are observed, indicating that the halide distribution remains substantially uniform. This suggests that PDADI effectively mitigates halide migration and preserves the optical properties of the film. The corresponding Δ*A* spectra for the passivated film, presented in Figure 2d, reinforce this conclusion. Both the negative and positive peaks are markedly smaller in magnitude compared to the control. Together, the absorption and PL results present compelling evidence that PDADI acts as an effective stabilizer of halide distribution in wide bandgap mixed halide perovskite films. By inhibiting the migration of halide ions and suppressing the formation of iodide-rich domains, PDADI helps preserve the optoelectronic uniformity of the perovskite layer. These effects are crucial for preserving photovoltaic performance and ensuring long-term device reliability, especially in tandem solar cell applications where wide bandgap absorbers are essential.

To evaluate the effect of PDADI passivation on the surface morphology and crystal growth behavior of FA_0.83_Cs_0.17_Pb­(I_0.6_Br_0.4_)_3_ perovskite films, SEM was conducted at different magnifications. Figure 3a and b show the surface morphology of the unpassivated (control) film, while Figure 3c and d corresponds to the PDADI passivated film. The control sample exhibits a compact, uniform polycrystalline structure with well-defined grain boundaries and consistent grain distribution. The absence of pinholes or large surface irregularities indicates high quality film formation. Upon PDADI treatment, the overall surface morphology remains similarly dense and uniform, confirming that the passivation process does not disrupt film continuity or induce detrimental morphological defects. Importantly, no significant change in the average grain size is observed between the passivated and unpassivated films, suggesting that PDADI does not induce bulk recrystallization or promote uncontrolled grain growth. Rather than altering the bulk structure, PDADI likely acts at the surface and grain boundaries, where it can effectively passivate defects without interfering with the underlying perovskite lattice. Particularly, a subtle but consistent difference is observed in the texture of the PDADI treated surface. The grains appear slightly smoother, and the grain boundaries are less sharply defined compared to the control. This smoother appearance may be attributed to the formation of a thin, conformal passivation layer at the perovskite surface. Such a layer could result from the interaction of −NH_3_^+^ groups of PDADI with undercoordinated Pb^2+^ ions at the grain boundaries or surfaces, leading to local surface reconstruction or chemical capping.^34^ This morphological signature is consistent with the presence of an ultrathin passivating interface, which can help mitigate surface defects, suppress nonradiative recombination, and reduce pathways for halide migration.

**Figure 3:**
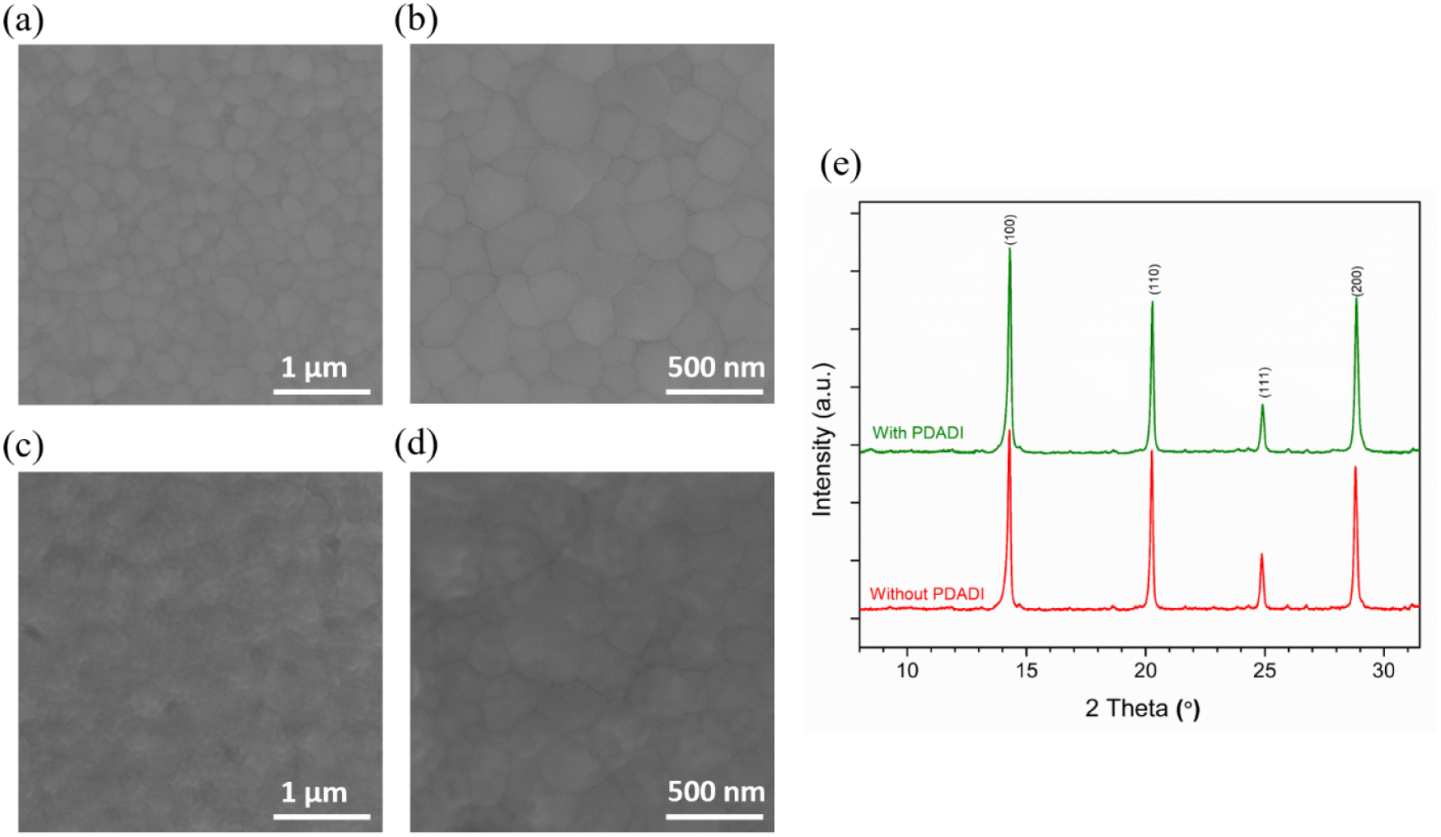
SEM images showing the surface morphology of (a, b) unpassivated (control) FA_0.83_Cs_0.17_Pb­(I_0.6_Br_0.4_)_3_ perovskite film at different magnifications and (c, d) PDADI passivated perovskite film at corresponding magnifications. (e) XRD patterns comparing the crystalline structure of unpassivated and PDADI passivated FA_0.83_Cs_0.17_Pb­(I_0.6_Br_0.4_)_3_ perovskite films.

The crystalline structure and phase purity of the FA_0.83_Cs_0.17_Pb­(I_0.6_Br_0.4_)_3_ perovskite films were further examined using XRD, as shown in Figure 3e. Both the control and PDADI passivated films exhibit distinct and well-defined diffraction peaks at approximately 2θ = 14.3°, 20.2°, 24.9°, and 28.8°, corresponding to the (100), (110), (111), and (200) planes of the FA_0.83_Cs_0.17_Pb­(I_0.6_Br_0.4_)_3_ perovskite lattice.^9,35,36^ These reflections confirm the successful formation of the desired 3D perovskite phase. Importantly, no additional peaks near 12.6°, which would indicate the presence of residual PbI_2_, are observed in either sample, confirming complete conversion of precursor materials and high phase purity.^37^ A key observation from the comparison is that PDADI passivation does not introduce any new diffraction peaks or structural distortions. The absence of features associated with secondary or low-dimensional perovskite phases suggests that PDADI does not alter the bulk crystal structure. This finding supports the SEM analysis, which indicated that the film morphology and grain structure remain largely unaffected at the bulk scale. However, a noticeable increase in the intensity of the primary diffraction peaks is observed in the PDADI treated film relative to the control. Although no significant peak shift is detected, this enhancement in peak intensity can be attributed to improved crystallinity, better molecular ordering, or reduced microstrain.^38,39^

To elucidate the chemical interactions responsible for the surface passivation behavior of PDADI, FTIR spectroscopy was employed. The full FTIR spectra of pure PbI_2_, pure PDADI, and the PDADI-PbI_2_ mixture are shown in Figure S2, while magnified views of the N–H/C–H stretching and bending regions are presented in Figure 4a and b, respectively. The FTIR spectrum of PDADI (red trace) shows distinct absorption bands in the 3150–2800 cm^–1^ range, primarily associated with N–H and C–H stretching vibrations from the terminal −NH_3_^+^ groups and the central aliphatic −CH_2_– chain.^40,41^ Additional features between 1600 and 400 cm^–1^ correspond to N–H bending and C–H bending modes.^41^ The spectrum of pure PbI_2_ (black trace) shows minimal absorption in these regions, as expected for a simple inorganic compound with limited IR activity. Upon mixing PDADI with PbI_2_ (blue trace), several of PDADI’s characteristic vibrational bands undergo noticeable shifts in position and intensity, indicating the formation of specific chemical interactions between the organic diammonium salt and PbI_2_. In the stretching region, PDADI exhibits clear peaks at 2987 cm^–1^ and 3028 cm^–1^, which are attributed to C–H stretching of the −CH_2_– groups and potentially overlapping N–H stretching of the terminal −NH_3_^+^ groups. In the PDADI-PbI_2_ mixture, a notable upshift of the N–H stretching mode is observed, with a new feature appearing at approximately 3097 cm^–1^. This shift indicates a strong perturbation of the N–H bonds, suggesting a more rigid or constrained environment due to coordination with Pb^2+^ ions.

**Figure 4:**
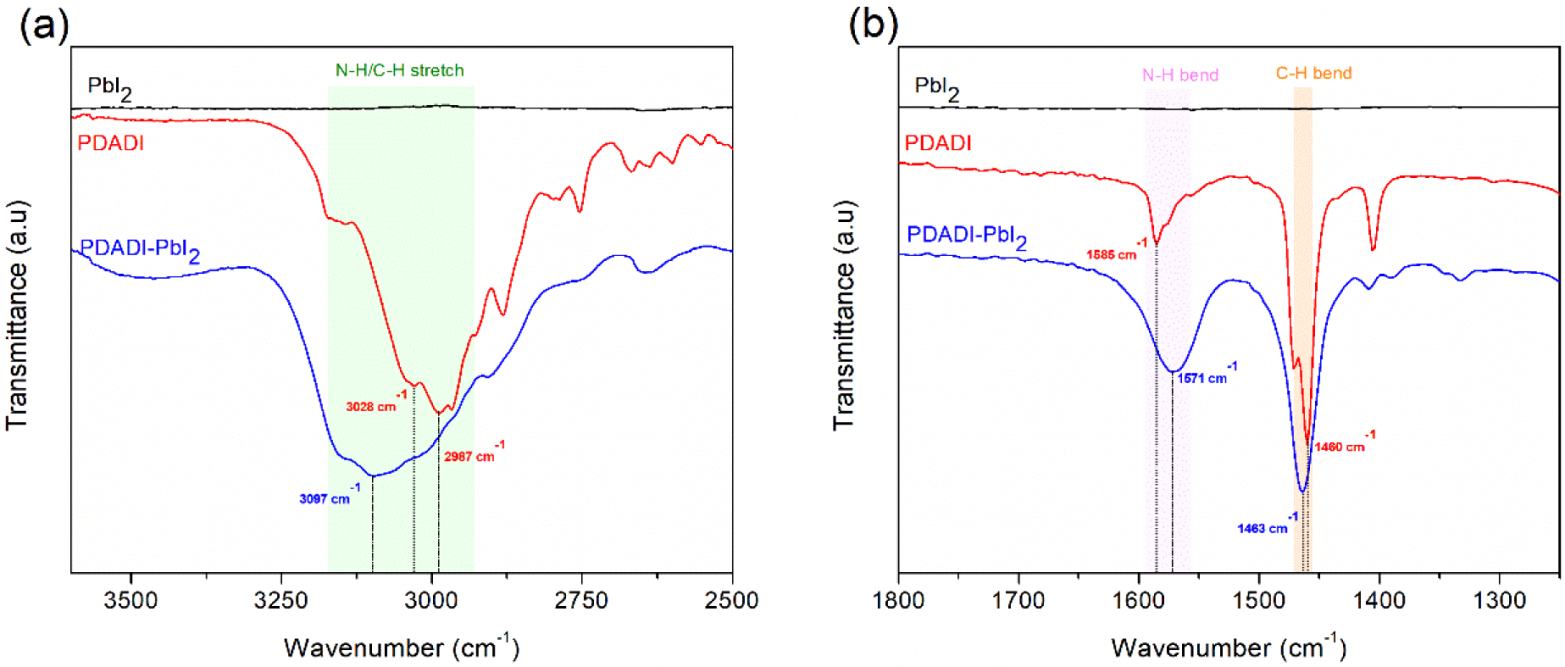
Magnified FTIR spectra of PbI_2_, PDADI, and PDADI-PbI_2_ mixture, focusing on specific vibrational regions: (a) N–H/C–H stretching region and (b) N–H and C–H bending region.

Such an upshift is often associated with hydrogen bonding or bidentate coordination, where the electron density from the −NH_3_^+^ groups is partially shared with Lewis acidic Pb^2+^ centers, effectively strengthening the N–H bond and increasing its vibrational frequency. The most compelling evidence for specific bonding lies in the bending region. The symmetric N–H bending mode for pure PDADI appears at 1585 cm^–1^, which undergoes a clear red shift to 1570 cm^–1^ in the PDADI-PbI_2_ mixture. A well-established signature of nitrogen to metal interaction, reflecting a reduction in the vibrational energy of the N–H bond due to its involvement in direct interaction with Pb^2+^ ions. Such a shift confirms that the terminal −NH_3_^+^ groups are the primary sites of binding. In contrast, the CH_2_ bending vibration shows only a minor shift from 1460 cm^–1^ in pure PDADI to 1463 cm^–1^ in the mixture, suggesting that the central aliphatic backbone remains largely unaffected. This subtle shift supports the conclusion that the coordination is localized at the amine termini, while the methylene bridge functions primarily as a structural spacer.

These collective vibrational shifts, specifically the upshift in N–H stretching and downshift in N–H bending along with the minimal change in CH_2_ vibrations, provide strong evidence that PDADI chemically interacts with the PbI_2_ surface via its terminal −NH_3_^+^ groups. The nature of this interaction is consistent with bidentate coordination or strong hydrogen bonding to undercoordinated Pb^2+^ atoms, which are dominant at the grain surfaces and boundaries of perovskite films. These Pb^2+^ surface sites are known to act as traps for photogenerated carriers and as initiation points for light-induced halide migration. By forming a conformal and chemically bonded surface layer, PDADI effectively passivates these defects, suppresses nonradiative recombination, and reduces ionic mobility, all of which contribute to halide distribution stability under illumination. This spectroscopic evidence strongly supports the improved structural stability observed in PDADI passivated perovskite films, aligning well with the minimal changes detected in both absorption and photoluminescence spectra under continuous illumination.

To fully understand the impact of PDADI passivation on the optoelectronic quality of the perovskite films, a comprehensive analysis of recombination kinetics and defect densities was performed using steady state PL, time-resolved PL and SCLC measurements. Figure 5a shows the steady state PL spectra of perovskite films without and with PDADI passivation. The PDADI passivated film exhibits a significantly higher PL intensity compared to the unpassivated control film. This substantial increase in PL intensity is a direct indication of reduced nonradiative recombination within the perovskite layer.^42^ Nonradiative recombination, primarily mediated by defects such as undercoordinated lead ions, halide vacancies, and grain boundaries, diminishes the number of excited charge carriers available for radiative emission.^14^ The enhanced PL intensity in the PDADI treated film therefore, signifies that these detrimental defect states have been effectively passivated, leading to an improved radiative recombination efficiency. Further quantitative insights into carrier dynamics were obtained from TRPL measurements, shown in Figure 5b. The decay dynamics were fitted using a biexponential decay function, which accounts for both fast (τ_1_) and slow (τ_2_) recombination processes.^43,44^ The fitted parameters and the calculated average PL lifetimes are summarized in Table S1. As clearly shown in Figure 5b, the PDADI passivated film exhibits a noticeably slower PL decay rate compared to the control. Quantitatively, the average PL lifetime (τ_avg_) for the control film is 172.6 ns, while the PDADI passivated film shows a significantly longer average lifetime of 218.9 ns. Both the fast (τ_1_) and slow (τ_2_) components of the decay are also improved in the PDADI treated sample. The fast component, often associated with rapid trap-assisted recombination at surfaces and grain boundaries, increases from 28.1 to 31.4 ns, indicating some passivation of these rapid pathways. More significantly, the slow component, often related to bulk recombination or defects away from highly active surfaces, increases from 216.9 to 263.0 ns. This collective increase in carrier lifetimes provides strong evidence for a substantial reduction in trap-assisted nonradiative recombination in the PDADI passivated perovskite films.^45^

**Figure 5:**
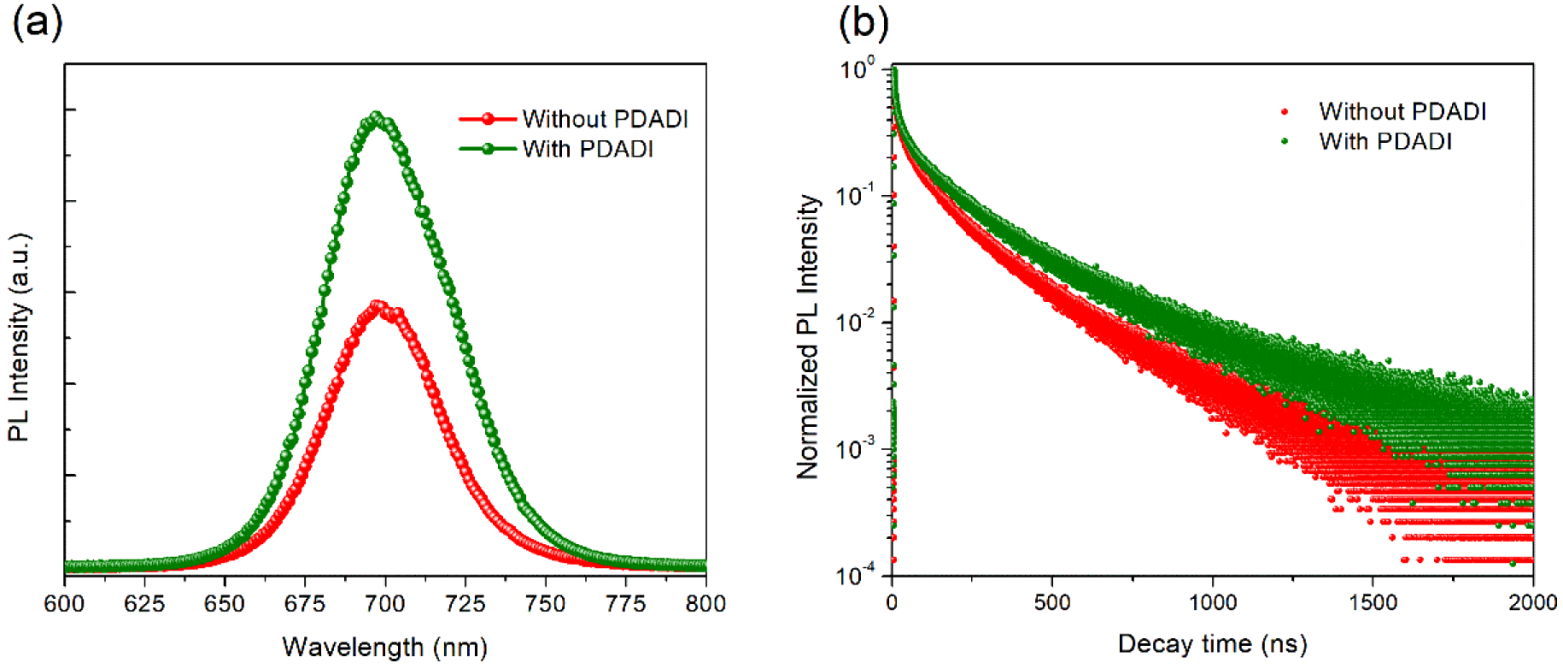
(a) Steady state PL spectra comparing the PL intensity of FA_0.83_Cs_0.17_Pb­(I_0.6_Br_0.4_)_3_ perovskite films without and with PDADI passivation. (b) Normalized time-resolved PL decay curves for the same films.

To quantitatively confirm the reduction in defect density, SCLC measurements were performed on electron-only devices with the structure ITO/SnO_2_/perovskite/with or without PDADI passivation/PCBM/Au under dark conditions. The current–voltage (*J*–*V*) characteristics for these devices are shown in Figure S3. The trap density was quantitatively determined from the trap-filled limit (TFL) voltage (*V*_TFL_) using the equation $N_{t}=\frac{{2\varepsilon }_{r}\varepsilon_{0}V_{\mathrm{TFL}}}{{\mathrm{eL}}^{2}}$ , where ε_r_ is the relative dielectric constant of the perovskite film, ε_0_ is the vacuum permittivity, *e* is the elementary charge, and *L* is the thickness of the perovskite film.^46–48^ Using this equation, the trap density is estimated to be 1.53 × 10^16^ cm^–3^ for the control device, which is significantly reduced to 1.05 × 10^16^ cm^–3^ for the PDADI passivated device. The SCLC measurements reveal a 31% reduction in the effective trap density for the PDADI-treated devices. This reduction suggests that PDADI not only passivates surface-level electronic traps but also likely penetrates into the upper grain boundaries of the polycrystalline film. Furthermore, the suppression of mobile ion migration by the bidentate diammonium anchors can lead to a decrease in the apparent space-charge density. As recently discussed in literature,^49^ effective surface passivation can mitigate the global trap-assisted recombination profile by stabilizing the interfaces where the majority of nonradiative losses occur, which is consistent with our observed increase in PL intensity and extension of carrier lifetimes.

This effective defect mitigation is a direct consequence of the molecular interactions revealed by FTIR spectroscopy, where diammonium groups of PDADI strongly coordinate to undercoordinated Pb^2+^ ions and potentially fill halide vacancies. By neutralizing these electronic traps, PDADI not only reduces nonradiative recombination but also enhances the quasi-Fermi level splitting, which is crucial for achieving high Voc.^50^ The prolonged carrier lifetimes ensure that charges have sufficient time to be efficiently extracted from the device, contributing positively to the FF. Therefore, the multifunctional role of PDADI in reducing defect density and enhancing charge carrier dynamics is essential for the overall improvement in the efficiency and stability of mixed halide perovskite solar cells. This fundamental improvement in electronic quality also plays a crucial role in preventing light-induced halide segregation, as defects often act as nucleation sites and pathways for ion migration.

To evaluate the impact of PDADI’s multifunctional role on device performance, PSCs were fabricated with a standard n-i-p planar architecture of ITO/SnO_2_/ FA_0.83_Cs_0.17_Pb­(I_0.6_Br_0.4_)_3_ perovskite/Spiro-MeOTAD/Au without and with PDADI passivation. Figure 6a presents the *J*–*V* characteristics of the best performing unpassivated and PDADI passivated PSCs. The inset table summarizes the key photovoltaic parameters for these champion devices. For the control device, the best PCE achieved is 14.11%, with Voc of 1.135 V, a Jsc of 18.07 mAcm^–2^ and a FF of 68.8%. However, the PDADI passivated PSC exhibits significantly enhanced performance. The best PDADI treated device achieves a remarkable PCE of 16.54%, representing a substantial improvement over the control. This enhancement is primarily driven by a notable increase in Voc to 1.243 V and an improved FF of 72.7%. A slight increase in Jsc to 18.30 mA cm^–2^ is also observed for the passivated device. As shown in Figure S4, the integrated Jsc derived from the EQE spectra are 16.87 mA cm^–2^ for the control device and 17.07 mA cm^–2^ for the PDADI treated device. These values closely match those obtained from the *J*–*V* measurements, confirming the reliability and consistency of the experimental results. Additionally, the degree of *J*–*V* hysteresis, an important indicator of ion migration and interfacial recombination, was significantly reduced in the PDADI treated devices. As shown in Figure S5 and Table S2, the hysteresis index (H-index) decreases from 9.6% (control) to 3.6% (PDADI), highlighting the improved charge extraction and reduced ionic mobility facilitated by PDADI passivation. To validate the consistency and reliability of the PDADI treatment, a statistical analysis was conducted for different devices produced under identical conditions. Figures 6b and S6 show box plots comparing the distributions of the photovoltaic parameters for unpassivated and PDADI passivated devices, while Table S3 summarizes their corresponding average values. The PDADI passivated devices not only show a significantly higher average PCE (15.73%) compared to the control (12.86%), but also exhibit much narrower standard deviations across all key parameters, indicating improved fabrication reproducibility and material uniformity. In particular, the average Voc and FF values are substantially higher in the PDADI passivated devices, while the lower spread in data highlights the enhanced reliability of the passivation process.

**Figure 6:**
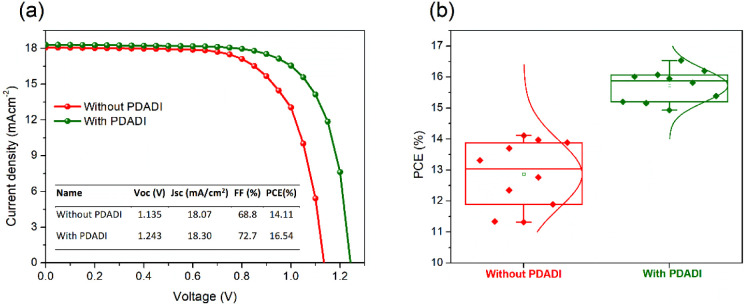
(a) Current density–voltage (*J*–*V*) characteristics of the best performing PSCs without and with PDADI passivation, measured under simulated AM 1.5G illumination (100 mW/cm^2^) and the inset table summarizes the key photovoltaic parameters for these champion devices. (b) Box plots showing the statistical distribution of PCE for multiple unpassivated and PDADI passivated devices.

The notable improvement in photovoltaic performance observed in PDADI treated devices is strongly supported by the fundamental material characterizations described in earlier sections. The significant increase in Voc from 1.135 V to 1.243 is directly attributed to the suppression of nonradiative recombination via defect passivation. Our steady state PL measurements showed a substantial increase in PL intensity (Figure 5a), and TRPL confirmed significantly longer carrier lifetimes in PDADI treated films (Figure 5b, Table S1). These optical findings are quantitatively supported by SCLC measurements, which revealed a substantial reduction in trap density upon PDADI passivation (Figure S3). This reduction leads to greater quasi-Fermi level splitting and consequently, higher Voc. The improvement in FF from 68.8% to 72.7% is attributed to decreased series resistance and enhanced charge transport. The significant reduction in *J*–*V* hysteresis from 9.6% to 3.6% further highlights the suppression of ion migration and charge accumulation. This directly links back to our initial observations from PL and absorption spectroscopy, where PDADI effectively limited light-induced halide segregation (Figures 1 and 2). FTIR spectroscopy (Figure 4) confirmed strong coordination between the diammonium groups of PDADI and undercoordinated Pb^2+^ ions, which stabilizes the lattice and prevents halide mobility. This molecular level stabilization translates to more stable and reproducible *J*–*V* curves, enhancing overall device reliability.

## 3 Conclusions

In this study, we demonstrate that 1,3-diaminopropane dihydroiodide (PDADI) serves as a highly effective and multifunctional passivation strategy for wide bandgap FA_0.83_Cs_0.17_Pb­(I_0.6_Br_0.4_)_3_ mixed halide perovskite solar cells. Our comprehensive analysis show that PDADI plays a crucial role in mitigating the persistent challenges of light-induced halide segregation and detrimental defect states, which are key limitations for these materials in tandem solar cell applications. Photoluminescence and UV–vis absorption measurements reveal that PDADI significantly suppresses halide segregation under illumination, maintaining halide distribution stability under illumination and stabilizing the bandgap. FTIR analysis confirms that the two −NH_3_^+^ groups of PDADI strongly coordinate with undercoordinated Pb^2+^ ions at the perovskite surface and grain boundaries, thereby stabilizing the lattice and inhibiting ion mobility. This effective chemical passivation leads to a substantial reduction in nonradiative recombination pathways and trap densities, resulting in enhanced PL intensity and significantly prolonged charge carrier lifetimes. These fundamental material improvements translated directly into superior photovoltaic device performance with PDADI passivated PSCs exhibiting significantly higher Voc and FF, ultimately achieving a notable increase in PCE. Furthermore, the pronounced reduction in *J*–*V* hysteresis highlights suppressed ion transport and improved charge carrier dynamics. Overall, the multifunctional role of PDADI in defect passivation, halide stabilization, and charge transport enhancement establishes a promising pathway for developing efficient, stable, and reproducible wide bandgap perovskite solar cells tailored for next generation tandem architectures.

## 4 Materials and Methods

### 4.1 Materials and Solvents

The ITO coated glass substrates (sheet resistance 7–10 ohm/sq) were obtained from MSC Supplies. Tin­(IV) oxide (SnO_2_) colloidal dispersion in water (15 wt %) was purchased from Alfa Aesar. Lead­(II) iodide (PbI_2_) was purchased from TCI Chemicals. Dimethyl sulfoxide (DMSO), *N*,*N*-dimethylformamide (DMF), lithium bis­(trifluoromethanesulfonyl)­imide (Li-TFSI), lead­(II) bromide (PbBr_2_), cesium iodide (CsI), formamidinium iodide (FAI), 4-*tert*-butylpyridine (t-BP), potassium chloride (KCl), anhydrous isopropanol (IPA), acetonitrile (ACN), and chlorobenzene (CB) were purchased from Sigma-Aldrich. 1,3-Diaminopropane dihydroiodide (PDADI) was obtained from Fluorochem. The hole transport material 2,2′,7,7′-tetrakis­(*N*,*N*-di-4-methoxyphenylamino)-9,9′-spirobifluorene (Spiro-MeOTAD) was obtained from Lumtec. [6,6]-Phenyl C61 butyric acid methyl ester (PCBM) was obtained from Special Carbon. All the chemicals were used as received without further purification.

### 4.2 Fabrication of FA_0.83_Cs_0.17_Pb­(I_0.6_Br_0.4_)_3_ Perovskite Solar Cells

The FA_0.83_Cs_0.17_Pb­(I_0.6_Br_0.4_)_3_ perovskite solar cells were fabricated with a standard n-i-p planar architecture. A schematic diagram of the device structure is provided in Figure S1. The device configuration consisted of ITO/SnO_2_/FA_0.83_Cs_0.17_Pb­(I_0.6_Br_0.4_)_3_/with and without PDADI passivation/Spiro-MeOTAD/Au. Each layer was sequentially deposited as described below, starting with the cleaning of the ITO-coated glass substrates.

#### 4.2.1 ITO Substrate Cleaning

The ITO-coated glass substrates underwent a thorough cleaning regimen to ensure optimal device performance. They were initially sonicated in a 2% solution of Hellmanex detergent diluted in deionized (DI) water for 30 min in an ultrasonic bath. Following this, they were rinsed with DI water and then further cleaned by sonication in acetone for 20 min, followed by another 20 min in isopropanol (IPA). After drying with a nitrogen flow, the substrates were exposed to UV-ozone treatment for 15 min to remove any remaining organic residues and to render the surface hydrophilic, promoting better film adhesion.

#### 4.2.2 Tin Oxide (SnO_2_) Electron Transport Layer Deposition

A 15 wt % SnO_2_ aqueous colloidal dispersion was diluted with deionized water at a 1:4 volume ratio. To this diluted solution, 2.5 mg of KCl was added per 1 mL to prepare a 2.5 mg/mL KCl-SnO_2_ mixture. The resulting solution was spin-coated onto cleaned ITO substrates at 4000 rpm for 30 s. The films were subsequently annealed at 150 °C for 30 min in air. After cooling down, the substrates were again treated with UV-ozone for 15 min before being transferred into a nitrogen-filled glovebox.

#### 4.2.3 Perovskite FA_0.83_Cs_0.17_Pb­(I_0.6_Br_0.4_)_3_ Film Deposition

The perovskite precursor solution was prepared by dissolving a precise mixture of components in a cosolvent system. Specifically, 142.7 mg FAI, 44.2 mg CsI, 184.4 mg PbI_2_, and 220.2 mg PbBr_2_ were dissolved in 1 mL of a solvent mixture comprising DMF and DMSO in a 4:1 (v/v) ratio. This solution was stirred constantly at 70 °C overnight to ensure complete dissolution and homogeneity. The perovskite solution was spin coated on SnO_2_ film at 1000 rpm for 10 s and 5000 rpm for 30 s. Importantly, 150 μL of CB antisolvent was dropped onto the spinning substrate 10 s before the end of the second spin-coating step. The samples were subsequently annealed at 100 °C for 30 min within the nitrogen-filled glovebox.

#### 4.2.4 PDADI Passivation Layer Deposition

For the PDADI passivation step, 1 mg of PDADI was dissolved in 1 mL of IPA. This solution was then spin-coated onto the perovskite layer at 4000 rpm for 30 s. Following deposition, the samples were annealed at 100 °C for 5 min to promote the passivation effect.

#### 4.2.5 Spiro-MeOTAD Hole Transport Layer Deposition

After the PDADI-passivated perovskite layer cooled to room temperature, a Spiro-MeOTAD layer was deposited by spin coating at 4000 rpm for 30 s. The Spiro-MeOTAD solution was prepared by dissolving the compound in chlorobenzene at a concentration of 72.3 mg/mL. To this, 28.8 μL of 4-*tert*-butylpyridine (*t*-BP) and 17.5 μL of a Li-TFSI solution (prepared by dissolving 520 mg of Li-TFSI in 1 mL of ACN) were added as dopants to enhance the hole conductivity of the Spiro-MeOTAD layer.

#### 4.2.6 Gold (Au) Electrode Deposition

Finally, after storing the fabricated devices overnight in a drybox, an 80 nm thick Au electrode was deposited by thermal evaporation under high vacuum. A shadow mask was used during this process to pattern the electrodes and define the active area of the solar cell.

### 4.3 Electron-Only Device Fabrication for SCLC Measurements

To measure the space charge limited current (SCLC) and assess defect densities, electron-only devices were fabricated with a specific architecture: ITO/SnO_2_/perovskite/with or without PDADI passivation/PCBM/Au. The procedures for depositing the SnO_2_ and FA_0.83_Cs_0.17_Pb­(I_0.6_Br_0.4_)_3_ perovskite layer, as well as the PDADI passivation layer (for relevant devices), were identical to those described for the full solar cells. The key difference was the replacement of the Spiro-MeOTAD hole transport layer with PCBM as the electron transport layer. The PCBM layer was formed by spin coating a 10 mg/mL solution of PCBM in chlorobenzene onto the perovskite film at 4000 rpm for 30 s. This film was then annealed at 100 °C for 10 min. An 80 nm Au top electrode was subsequently deposited as described for the full solar cells.

### 4.4 Materials and Device Characterization

The morphology of the perovskite films was examined using a field emission scanning electron microscope (FE-SEM) (Tescan MAIA) operated at an accelerating voltage of 5 kV. UV–vis absorption spectra were recorded with a Bentham system, which included a TMC 300 monochromator and a 50 W tungsten halogen lamp. Steady state photoluminescence (PL) spectra were recorded with a Cary Eclipse fluorescence spectrophotometer under 300 nm excitation. Time-resolved PL decay measurements were performed using a FluoTime 200 system (PicoQuant GmbH), excited by a 442 nm picosecond pulsed laser. A Rigaku SmartLab diffractometer equipped with a 9 kW copper rotating anode X-ray source (Cu Kα radiation, λ = 0.15418 nm) was used to obtain the XRD patterns in Bragg–Brentano geometry. Fourier-transform infrared spectroscopy (FTIR) measurements were carried out using a VERTEX 80 Bruker FTIR spectrophotometer. The current density–voltage (*J*–*V*) characteristics were measured under simulated AM 1.5G solar illumination (100 mW/cm^2^) using the LED solar simulator (Wavelabs Solar Metrology Systems) and a computer-controlled Keithley 2400 source meter. A metal mask defining an active area of 0.09 cm^2^ was used during *J*–*V* measurements. External quantum efficiency (EQE) measurements were carried out with a Bentham system consisting of a TMC 300 monochromator, 50 W tungsten halogen lamp, and a lock-in amplifier, using a calibrated silicon photodiode as the reference. The current–voltage characteristics of the electron-only devices were measured in the dark using the same Keithley 2400 source meter. All measurements were conducted at room temperature under ambient conditions without encapsulation.

## Supplementary Material



## Notes

Schematic device structure; full FTIR spectra, summary of the fit and calculated parameters of the TRPL spectra; the dark *I*–*V* curves of the electron-only devices; EQE spectra; *J*–*V* curves under reverse and forward scans; statistical distribution box plots of photovoltaic parameters, the average photovoltaic parameters (PDF)
