# Supplementary material for: Stabilizing Halide Distribution in Mixed Halide Perovskites through Diammonium-Based Passivation
Source: ACS Omega. 2026 Mar 16;11(12):19903–13. doi: 10.1021/acsomega.6c01620 (PMC13044657; doi:10.1021/acsomega.6c01620)
Supplement: Supplementary file 1 [file ao6c01620_si_001.pdf]

Supporting Information:

Stabilizing halide distribution in mixed halide perovskites through diammonium based passivation

Amalraj Peter Amalathas[1,2*], Saisankar Sunthareswaran[1], Neda Neykova[2,3], Lukáš Horák[4], and Jakub Holovský[2,3*]

[1]*Department of Physics, Faculty of Science, University of Jaffna, Jaffna 40000, Sri Lanka*

[2] *SOL-MAT lab (Solar cell material laboratory), Faculty of Electrical Engineering, Czech Technical University in Prague, Technická 2, 166 27 Prague, Czech Republic*

[3] *Institute of Physics, Czech Academy of Sciences, v. v. i., Cukrovarnická 10, 162 00 Prague, Czech Republic*

[4] *Department of Condensed Matter Physics, Faculty of Mathematics and Physics, Charles University, Ke Karlovu 5, 12116, Prague 2, Czech Republic*

* *Corresponding author email: amalraj@univ.jfn.ac.lk; holovsky@fzu.cz*

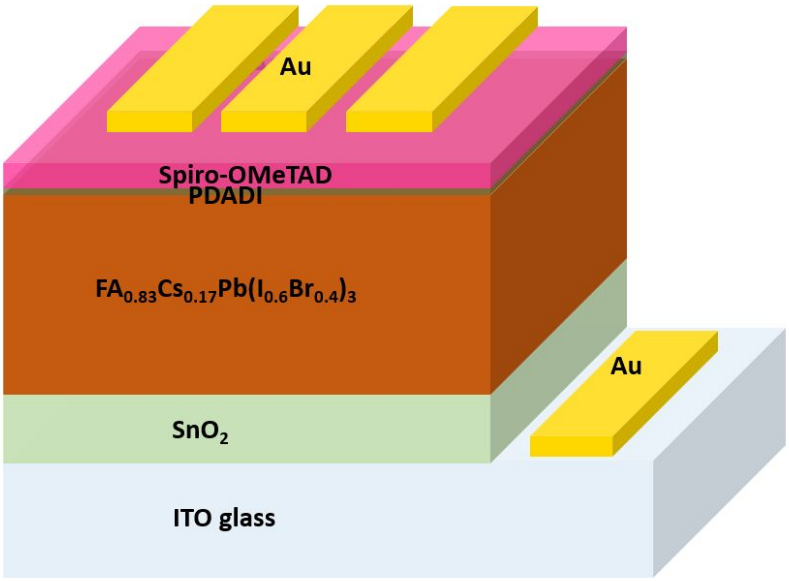

Figure S1: Schematic device structure of planar perovskite solar cells with the device configuration of ITO/SnO_2/ $FA_{0.83}Cs_{0.17}Pb(I_{0.6}Br_{0.4})_3$ perovskite/ PDADI/Spiro-OMeTAD/Au

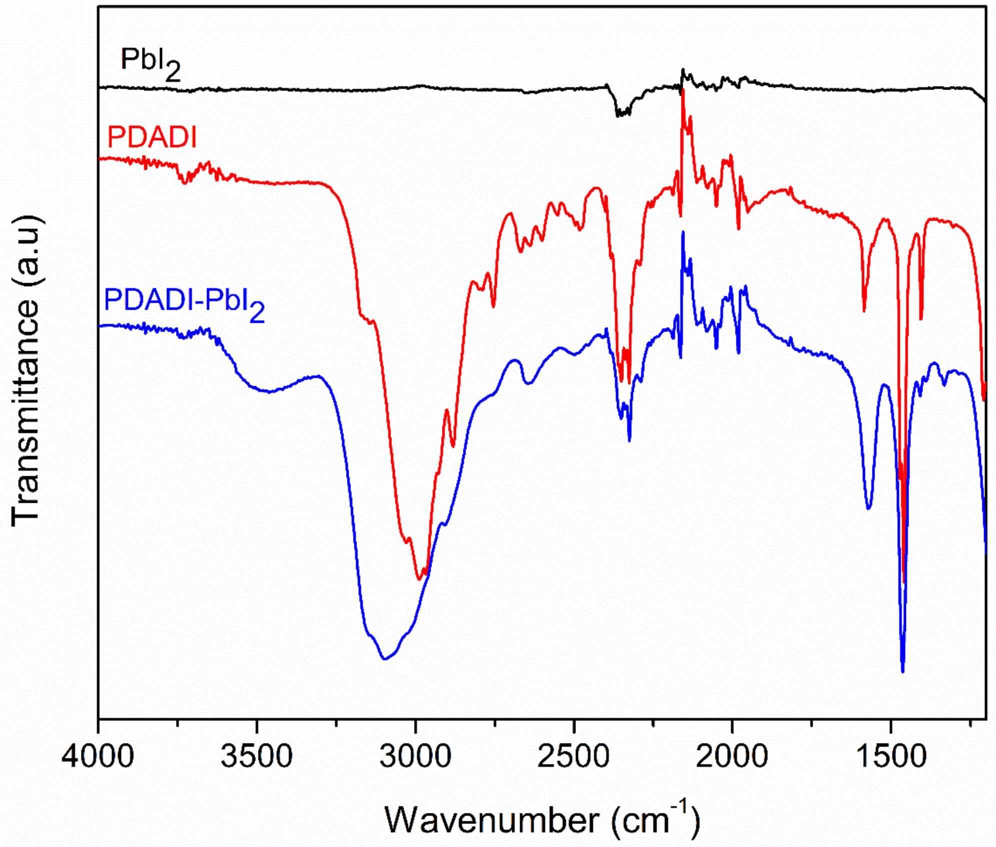

Figure S2: Full FTIR spectra of pure lead iodide (PbI_2), pure 1,3-propanediamine dihydroiodide (PDADI), and their mixture (PDADI-PbI_2).

The carrier lifetime is obtained by fitting the PL transient decays with a bi-exponential decay function as follows

$$f(t) = A_1 exp^{(-\frac{t}{\tau_1})} + A_2 exp^{(-\frac{t}{\tau_2})} + B$$

where A_1 and A_2 represent the time-independent decay amplitudes, B is a constant, and τ_1 and τ_2 are the fast and slow decay time, respectively. The weighted-average lifetime (τ_{avg}) is calculated from the fit curve parameters according to the following equation.

$$\tau_{avg} = \frac{A_1 {\tau_1}^2 + A_2 {\tau_2}^2}{A_1 \tau_1 + A_2 \tau_2}$$

Table S1 Bi-exponential fitted parameters and average PL lifetimes for perovskite films.

Sample name	A_1(%)	τ_1 (ns)	A_2(%)	τ_2 (ns)	Average τ (ns)
Without PDADI	70.31	28.09	29.69	216.92	172.60
With PDADI	66.35	31.41	33.65	263.04	218.90

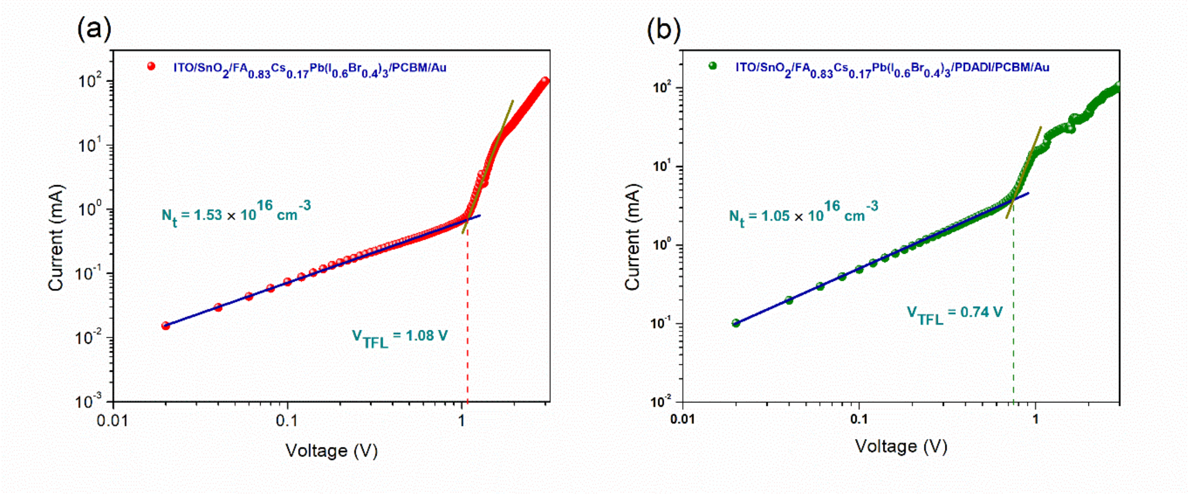

Fig. S3 Current-voltage (J-V) characteristics of electron-only devices with the structure ITO/SnO_2/$FA_{0.83}Cs_{0.17}Pb(I_{0.6}Br_{0.4})_3$ perovskite /with or without PDADI passivation/PCBM/Au: (a) without PDADI passivation and (b) with PDADI passivation. The trap-filled limit voltage (V_{TFL}) and corresponding trap density (N_t) are indicated for each device.

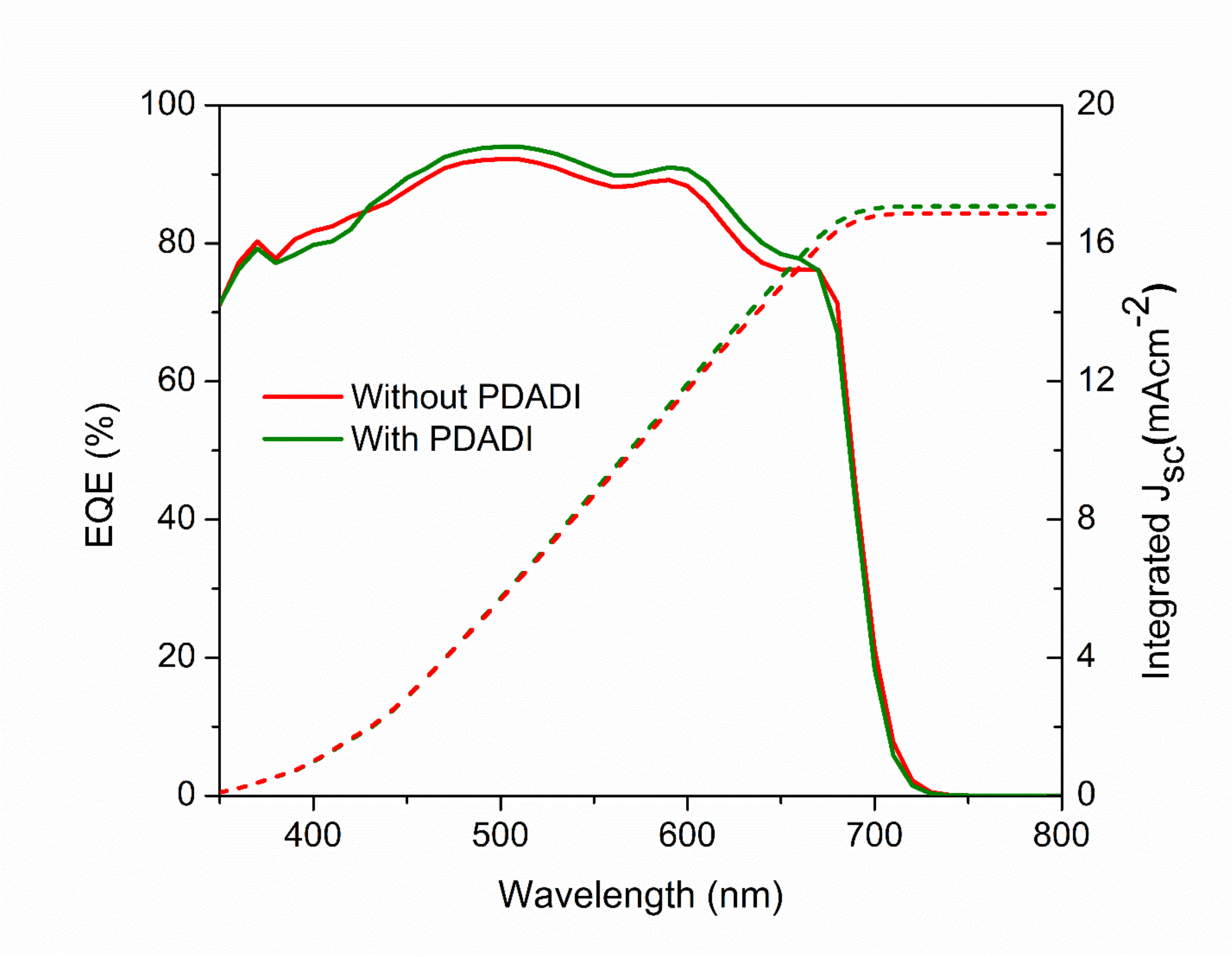

Fig. S4 The external quantum efficiency (EQE) spectra and integrated J_{SC} for the best performing perovskite solar cells prepared without and with PDADI passivation.

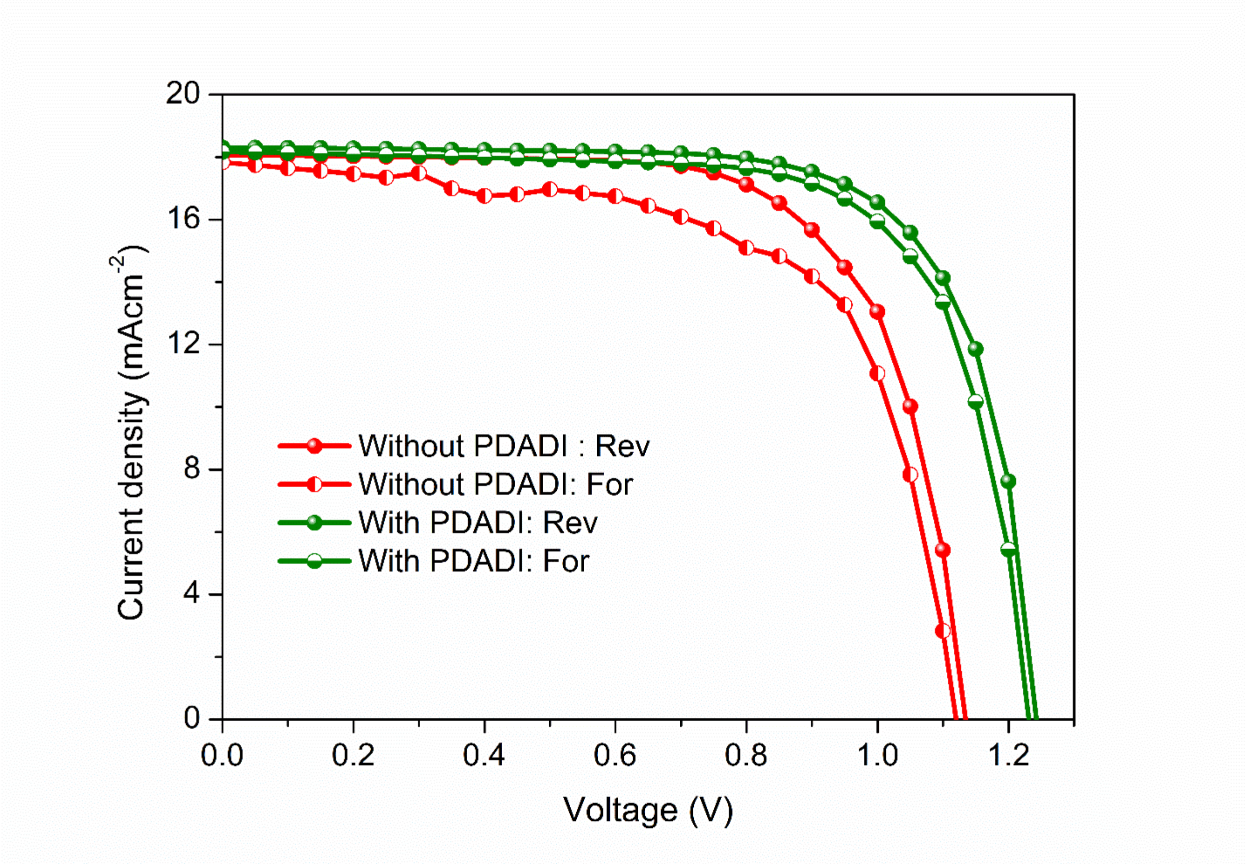

Fig. S5 Current density–voltage (J–V) curves under reverse and forward scans for the perovskite solar cells prepared with and without PDADI passivation.

Table S2 Summary of photovoltaic parameters from reverse and forward scans and hysteresis index (H-index) calculation.

Name	Voc (V)	Jsc (mA/cm^2)	FF (%)	PCE(%)	H-Index (%)
Without PDADI: Reverse	1.135	18.07	68.8	14.11	9.6
Without PDADI: Forward	1.120	17.83	63.9	12.76	
With PDADI: Reverse	1.243	18.30	72.7	16.54	3.6
With PDADI: Forward	1.231	18.17	71.3	15.95	

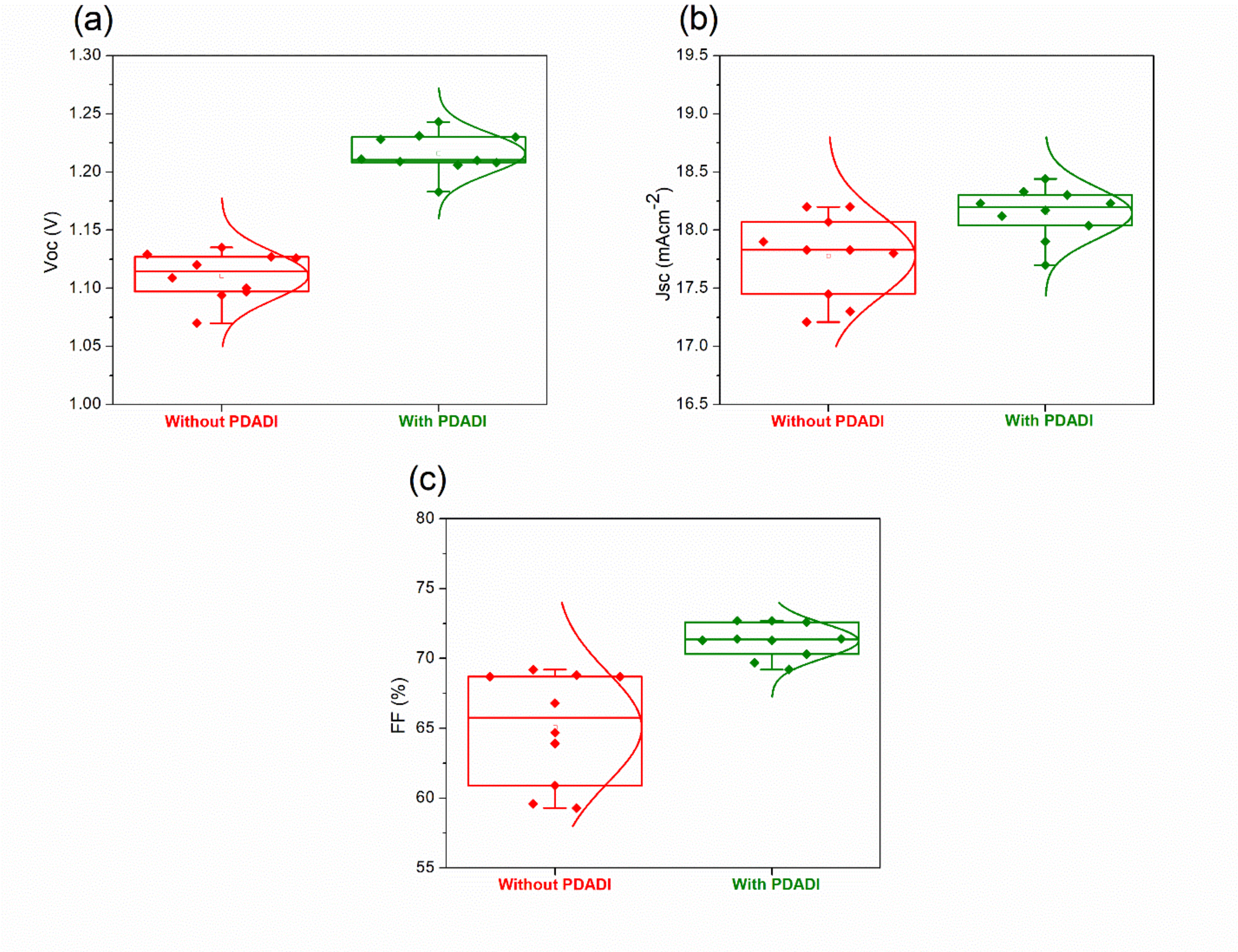

Fig. S6 Box plots showing the statistical distribution of (a) Voc, (b) Jsc and (c) FF for multiple unpassivated and PDADI passivated perovskite solar cells (Statistics based on 10 devices).

Table S3 Summary of the average photovoltaic parameters of the PSCs without and with PDADI passivation.

Name	Voc (V)	Jsc (mA/cm^2)	FF(%)	PCE(%)	Average HI(%)
Without PDADI	1.111 ± 0.020	17.78 ± 0.35	65.1 ± 4	12.86 ± 1.09	10.9 ± 1.2
With PDADI	1.216 ± 0.017	18.15 ± 0.22	71.3 ± 1.2	15.73 ± 0.53	4.1 ± 0.6
